# From Cholinergic Crisis to Anticholinergic Delirium: When the Antidote Mimics the Poison

**DOI:** 10.7759/cureus.115146

**Published:** 2026-08-25

**Authors:** Venkata Yashashwini Maram Reddy, Charan Teja Thummuru, Shreya Rani, Venkata Sai Rithin Maramreddy, Krishna Reddy Maramreddy

**Affiliations:** 1 Medicine and Surgery, Guntur Medical College, Guntur, IND; 2 Internal Medicine, Gandhi Medical College & Hospital, Hyderabad, IND; 3 General Medicine, Indira Gandhi Institute of Medical Sciences, Patna, IND; 4 Medicine, Narayana Medical College, Nellore, IND; 5 Obstetrics and Gynaecology, Sai Nursing Home, Darsi, IND

**Keywords:** anticholinergic toxicity, atropine-induced delirium, cholinergic crisis, monocrotophos poisoning, organophosphorus poisoning

## Abstract

Organophosphorus pesticide poisoning remains a major public health concern due to widespread agricultural use and easy availability of these compounds. Atropine is the cornerstone of treatment for life-threatening muscarinic manifestations. However, repeated or prolonged administration can cause central anticholinergic toxicity, which may closely resemble the neurological effects of organophosphorus poisoning and delay the correct diagnosis. We report a 26-year-old woman who developed severe cholinergic toxicity after intentionally ingesting monocrotophos and was treated with mechanical ventilation, pralidoxime, and atropine. After 48 hours of atropine therapy, she developed acute agitation, hallucinations, disorientation, and peripheral anticholinergic findings despite resolution of her initial cholinergic symptoms. Other metabolic, infectious, and toxicological causes were not identified. Her symptoms resolved after atropine was discontinued, without cholinergic rebound. This case emphasizes the importance of distinguishing atropine-induced delirium from ongoing organophosphorus central nervous system (CNS) toxicity. Regular clinical reassessment and symptom-guided atropine titration may prevent unnecessary investigations, inappropriate treatment, and avoidable anticholinergic complications.

## Introduction

Organophosphorus pesticide poisoning persists as a critical global health challenge, most notably across agricultural landscapes in developing and middle-income nations. While the exact epidemiological impact is obscured by frequent underreporting, self-poisoning with these insecticides has historically represented a major portion of pesticide-related mortality, accounting for over 100,000 deaths annually [1]. In India, the burden is particularly profound due to the ready availability of highly lethal compounds and their common use in intentional ingestions, frequently involving young adults and rural residents [1,2]. 

Mechanistically, these compounds work by inhibiting acetylcholinesterase, which triggers a pathological accumulation of acetylcholine at muscarinic, nicotinic, and central nervous system synapses. Clinical presentations often include severe bronchorrhea, bronchospasm, bradycardia, and gastrointestinal distress, alongside fasciculations, muscle weakness, and psychiatric disturbances ranging from seizures to delirium [3]. Intravenous atropine remains the cornerstone of management for muscarinic toxicity, administered in rapidly doubling doses until pulmonary secretions are suppressed and bronchoconstriction resolves. Subsequently, maintenance therapy is titrated based on the presence or absence of recurring cholinergic features [4,5]. Although rapid atropinization is essential in severe poisoning, the optimal loading and maintenance regimens remain incompletely standardized. Comparative evaluations of published protocols have demonstrated substantial variation in recommended atropine doses and the time required to achieve atropinization, reinforcing the need for frequent, clinically guided dose adjustment [6-9].

Despite its therapeutic necessity, intensive or prolonged atropine therapy can precipitate central antimuscarinic delirium. This iatrogenic state may present as profound agitation, hallucinations, incoherent speech, and disorientation, typically accompanied by peripheral anticholinergic signs such as mydriasis, dry mucosa, urinary retention, and tachycardia [1]. Distinguishing this from the underlying organophosphorus toxicity, hypoxia, or sepsis is complex, as these conditions often share similar neuropsychiatric features. Consequently, delirium in this setting is frequently multifactorial, arising from the interplay between the pesticide and the antidote [4]. 

Atropine-induced delirium can mimic the central nervous system (CNS) manifestations of organophosphorus poisoning and may be easily missed. This case highlights the importance of recognizing the shift from cholinergic toxicity to an anticholinergic clinical picture, as failure to identify atropine-associated delirium may lead to unnecessary continuation or escalation of atropine therapy.

## Case presentation

A 26-year-old woman was brought to the emergency department approximately six hours after the intentional ingestion of a monocrotophos-containing pesticide in a suicide attempt. The episode occurred in the setting of significant psychosocial stressors, including recent loss of employment and housing instability. She was subsequently found by her parents and brought to the hospital for further management.

Following ingestion, she developed multiple episodes of vomiting, excessive salivation, lacrimation, abdominal pain, profuse sweating, urinary and fecal incontinence, and progressively worsening difficulty in breathing. A characteristic garlic-like odor was noted on her breath.

Her medical history was significant for hypothyroidism, for which she was receiving levothyroxine 50 μg once daily, and a previous ectopic pregnancy. She was also taking multivitamin supplements. There was no reported history of alcohol consumption, smoking, recreational drug use, or other substance exposure.

On admission, the patient was drowsy but responsive to verbal commands and was disoriented to time, place, and person, with a Glasgow Coma Scale score of 13/15. Her heart rate was 46 beats/min, blood pressure was 80/40 mmHg, respiratory rate was 6 breaths/min, oxygen saturation was 80% on room air, and body temperature was 96°F. She weighed 60 kg and was 156 cm tall, corresponding to a body mass index of 24.7 kg/m². Her pupils were bilaterally pinpoint, with marked salivation and frothing at the angles of the mouth. Cardiovascular examination revealed bradycardia with normal heart sounds. Respiratory examination demonstrated hypopnea, reduced chest expansion, diminished bilateral air entry, and use of accessory muscles, without clinically evident cyanosis. Wheezing was present with reduced bilateral breath sounds on auscultation. Motor examination demonstrated reduced tone and generalized weakness; she moved all four limbs in response to commands. Deep-tendon reflexes were diminished bilaterally. Abdominal examination revealed generalized tenderness without distension or visible abnormality, with hyperactive bowel sounds. Urinary and fecal incontinence had occurred before presentation.

Laboratory and cardiopulmonary investigations performed at admission and during the repeat diagnostic evaluation are summarized in Table 1.

**Table 1:** Admission and repeat diagnostic evaluation ABG: arterial blood gas; AST: aspartate aminotransferase; ALT: alanine aminotransferase; PaCO₂: partial pressure of arterial carbon dioxide; PaO₂: partial pressure of arterial oxygen; HCO₃: bicarbonate; β-hCG: beta-human chorionic gonadotropin. An em dash indicates that the investigation was not performed, not repeated, or not reported.

Category	Investigation	Admission result	Reference range	Initial interpretation	Repeat result	Repeat findings
Complete blood count	Hemoglobin	8.0 g/dL	12.0–16.0 g/dL	Moderate anemia	8.1 g/dL	No major interval change
	Total leukocyte count	12,000 cells/mm³	4,000–11,000 cells/mm³	Mild leukocytosis	10,200 cells/mm³	Improved
	Neutrophils	80%	40–70%	Neutrophilia	—	Not repeated
	Lymphocytes	15%	20–40%	Relative lymphopenia	—	Not repeated
	Platelet count	150,000/mm³	150,000–450,000/mm³	Lower limit of normal	165,000/mm³	No significant abnormality
Glucose and electrolytes	Random blood glucose	50 mg/dL	70–140 mg/dL	Hypoglycemia	101 mg/dL	Corrected
	Serum sodium	130 mmol/L	135–145 mmol/L	Mild hyponatremia	136 mmol/L	Normalized
	Serum potassium	3.0 mmol/L	3.5–5.0 mmol/L	Hypokalemia	3.6 mmol/L	Normalized
	Serum chloride	90 mmol/L	98–106 mmol/L	Hypochloremia	—	Not repeated
	Serum bicarbonate	20 mmol/L	22–28 mmol/L	Reduced	—	Assessed in repeat ABG.
	Serum calcium	8.0 mg/dL	8.5–10.5 mg/dL	Mild hypocalcemia	10.0 mg/dL	No significant abnormality
	Serum magnesium	2.0 mg/dL	1.7–2.4 mg/dL	Normal	2.0 mg/dL	No significant abnormality
	Serum phosphate	3.0 mg/dL	2.5–4.5 mg/dL	Normal	—	Not repeated
Renal function	Blood urea nitrogen	20 mg/dL	7–20 mg/dL	Upper limit of normal	16 mg/dL	No significant deterioration
	Serum creatinine	1.4 mg/dL	0.5–1.1 mg/dL	Mild elevation	0.9 mg/dL	Improved
Liver function	AST	50 U/L	10–40 U/L	Mild elevation	35 U/L	Improved
	ALT	55 U/L	7–35 U/L	Mild elevation	34 U/L	Improved
	Alkaline phosphatase	140 U/L	44–147 U/L	Within reference range	—	Not repeated
	Total bilirubin	1.0 mg/dL	0.2–1.2 mg/dL	Normal	0.8 mg/dL	No major interval abnormality
	Serum albumin	3.0 g/dL	3.5–5.0 g/dL	Mildly reduced	3.1 g/dL	No major interval change
Muscle and coagulation profile	Creatine kinase	150 U/L	30–170 U/L	Normal	—	Not repeated
	Prothrombin time	11 seconds	11–13.5 seconds	Normal	—	Not repeated
	International normalized ratio	1.1	0.8–1.2	Normal	—	Not repeated
Thyroid profile	Thyroid-stimulating hormone	0.6 mIU/L	Approximately 0.4–4.0 mIU/L	Normal	—	Not repeated
Inflammatory marker	C-reactive protein	—	—	Not assessed at admission	4 mg/dL	No significant elevation reported
Toxicology assessment	Serum pseudocholinesterase	2,000 U/L	4,620–11,500 U/L	Reduced	—	Not repeated
	Red-cell acetylcholinesterase	8,000 IU/L	11,188–16,698 IU/L	Reduced	—	Not repeated
	Urine toxicology screen	Negative for common co-ingestants	—	Negative	—	No additional agent identified
	Review for additional medications or co-ingestants	—	—	—	No additional agent identified	No additional offending medication identified
	Alcohol and recreational-drug history	Negative	—	No reported use	Negative	No history or evidence of recent use
Arterial blood gas	pH	7.18	7.35–7.45	Acidemia	Normal	Improved
	PaCO₂	55 mmHg	35–45 mmHg	Hypercapnia	Normal	Improved
	PaO₂	48 mmHg	80–100 mmHg	Severe hypoxemia	Normal	Improved
	HCO₃⁻	19 mmol/L	22–26 mmol/L	Reduced	Normal	Improved
	Lactate	4.2 mmol/L	0.5–2.0 mmol/L	Elevated	—	Not repeated
Urine and pregnancy testing	Urinalysis	No significant abnormality	—	Unremarkable	—	Not repeated
	Urine or serum β-hCG	Negative	Negative	Pregnancy excluded	—	Not repeated
Cardiac assessment	Electrocardiogram	Sinus bradycardia at 46 beats/min without acute ischemic or conduction abnormalities		Consistent with cholinergic toxicity	Sinus tachycardia at 108 beats/min	No acute ischemic or conduction abnormalities
Pulmonary assessment	Oxygen saturation	80% on room air	95–100%	Hypoxemia	97% on room air	Improved compared with admission
	Chest radiograph	No acute cardiopulmonary abnormality	—	No infiltrate, edema, or aspiration at presentation	No acute cardiopulmonary abnormality	No new infiltrate or acute cardiopulmonary abnormality

Management 

The patient underwent immediate airway suctioning, oxygen supplementation, endotracheal intubation, and mechanical ventilation because of severe bronchorrhea, hypoventilation, and hypoxemia. Intravenous atropine was initiated with a 2 mg bolus, followed by rapidly escalating doses of 4 mg, 8 mg, and 16 mg at five-minute intervals until bronchial secretions cleared, bilateral air entry improved, and bradycardia resolved. A cumulative atropine loading dose of 30 mg was administered, followed by a continuous infusion of 6 mg/hour. The infusion was subsequently reassessed according to bronchial secretions, bilateral air entry, oxygenation, heart rate, and recurrence of cholinergic manifestations. Pralidoxime was given as a 2 g intravenous loading dose over 30 minutes, followed by continuous infusion at 500 mg/hour for 48 hours. She additionally received intravenous crystalloids, dextrose for hypoglycemia, potassium replacement, gastric protection, and norepinephrine for persistent hypotension.

Clinical progression 

After approximately 48 hours of therapy and a cumulative atropine exposure of 318 mg, she developed abrupt behavioral and neuropsychiatric changes characterized by marked restlessness, agitation, insomnia, irrelevant and incoherent speech, disorganized behavior, and visual hallucinations. She became increasingly uncooperative and was disoriented to time, place, and person, despite improvement in her initial cholinergic manifestations. At the onset of these symptoms, bronchial secretions had markedly decreased, while examination demonstrated dilated pupils, dry oral mucosa, warm dry skin, reduced bowel sounds, and urinary retention, raising concern for central anticholinergic toxicity. A repeat diagnostic evaluation was performed and is summarized in Table 1.

In view of the temporal relationship with atropine exposure and exclusion of other causes, atropine-associated delirium was suspected. The atropine infusion was discontinued, and supportive care was continued with intravenous hydration, environmental reorientation, and short-term pharmacological control of severe agitation as required. Her symptoms improved within 24 hours and resolved completely within 48 hours, without recurrence of bronchorrhea or bradycardia. At discharge, she was conscious, cooperative, fully oriented, and free of hallucinations, agitation, or other behavioral disturbances. Following psychiatric evaluation and suicide-risk assessment, she was advised regular psychiatric follow-up, medication adherence where indicated, and receive close family supervision. 

## Discussion

Atropine is central to the treatment of organophosphorus poisoning. However, the repeated or prolonged doses required in severe poisoning may also produce central antimuscarinic toxicity. In this patient, the development of acute confusion and behavioral changes during atropine therapy created an important diagnostic dilemma: persistent central cholinergic toxicity from organophosphorus poisoning versus iatrogenic central anticholinergic delirium from atropine.

Organophosphorus compounds inhibit acetylcholinesterase, leading to excessive acetylcholine at muscarinic, nicotinic, and central nervous system synapses [1,3]. Atropine counteracts the muscarinic effects of this excess acetylcholine and is particularly important for controlling bronchorrhea, bronchospasm, bradycardia, and impaired oxygenation [1,4]. However, because atropine crosses the blood-brain barrier, excessive doses can block central muscarinic receptors and cause confusion, agitation, hallucinations, disorientation, and delirium [5-7]. The risk of central toxicity is influenced by the intensity and duration of atropine exposure and by individual susceptibility; therefore, cumulative dose alone should not be regarded as a definitive toxicity threshold [10,11].

The main difficulty is that organophosphorus poisoning itself can cause similar neurological symptoms, including agitation, confusion, seizures, reduced consciousness, and coma [3,4]. Delirium in these patients may also result from hypoxia, infection, metabolic disturbances, alcohol or other co-ingestants, withdrawal, or complications of intensive care [4]. Therefore, atropine-induced delirium is primarily recognized by the timing of symptoms and the shift from cholinergic to anticholinergic findings [4,7].

Comparison of organophosphorus and atropine toxicity

The key clinical differences between organophosphorus toxicity and atropine toxicity are summarized in Table 2.

**Table 2:** Clinical comparison of organophosphorus and atropine toxicity

Features	Organophosphorus toxicity	Atropine toxicity
Mechanism	Acetylcholinesterase inhibition causes excessive acetylcholine activity [1,3].	Muscarinic receptor blockade causes peripheral and central anticholinergic effects [4,7].
Mental status	Confusion, agitation, seizures, reduced consciousness, or coma [3,4].	Agitation, disorientation, hallucinations, incoherent speech, and delirium [6,7].
Pupils	Miosis is common [3,4].	Mydriasis may occur [6,7].
Secretions	Salivation, sweating, and bronchorrhea are common [3,4].	Dry mouth, dry skin, and reduced respiratory secretions are common [6,7].
Respiratory findings	Bronchorrhea, bronchospasm, and respiratory failure may occur [1,3,11].	Bronchial secretions are reduced [4,7,11].
Heart rate	Bradycardia, but tachycardia may also occur [3,4].	Tachycardia is common but is not diagnostic on its own [4,7].
Gastrointestinal and urinary findings	Vomiting, diarrhea, abdominal cramps, and urination [3].	Reduced bowel sounds, constipation, and urinary retention [6,7].
Neuromuscular findings	Fasciculations, weakness, and respiratory muscle paralysis [1,3].	Fasciculations and nicotinic muscle weakness are not typical [4].
Response to atropine	Cholinergic and respiratory symptoms improve [1,4].	Delirium generally improves after atropine is reduced or temporarily withheld [5–7].

Several findings in our patient favored atropine-induced delirium. The confusion and behavioral changes developed after repeated atropine administration, while the initial cholinergic and respiratory symptoms had improved. Peripheral anticholinergic findings were also present. No metabolic, infectious, structural, or co-ingestant cause was identified, and the symptoms improved after atropine was reduced or temporarily withheld. Taken together, these findings made ongoing organophosphorus CNS toxicity less likely and supported atropine-associated delirium as the most likely clinical explanation [4,6,7].

A similar presentation was reported by Moudgil et al., in which a patient developed delirium, hallucinations, and altered behavior during atropine treatment for organophosphorus poisoning [6]. As in the present case, the neuropsychiatric manifestations developed during atropine therapy and improved after modification of atropine exposure. Our patient additionally demonstrated a clear transition from initial cholinergic manifestations to peripheral anticholinergic findings, including mydriasis, dry mucosa, warm dry skin, reduced bowel sounds, and urinary retention, further supporting the clinical distinction between the two toxidromes.

Careful atropine titration can reduce this risk. Perera et al. reported delirium in 17% of patients receiving ad hoc atropine dosing compared with only 1% of those managed using a structured, clinically titrated regimen. Hallucinations were also more frequent with ad hoc dosing than with titrated dosing, occurring in 35% and 1% of patients, respectively [5]. This difference highlights the importance of regular clinical reassessment rather than continuing a fixed or empirically selected atropine dose.

Atropine should be titrated mainly to the resolution of clinically significant bronchorrhea, bronchospasm, impaired oxygenation, hypotension, and dangerous bradycardia [1,4]. Heart rate, pupil size, and mucosal dryness should not be used alone to guide treatment, as these findings do not reliably show whether adequate pulmonary atropinization has been achieved [4]. After stabilization, the maintenance infusion should be reviewed frequently and adjusted according to the return of cholinergic symptoms or the appearance of anticholinergic toxicity [4,5].

Future relevance 

Greater awareness of atropine-induced delirium may help clinicians recognize the condition earlier, reduce unnecessary investigations, and avoid inappropriate psychiatric or neurological treatment. Prospective studies are needed to evaluate standardized atropine maintenance and de-escalation strategies and to develop validated clinical criteria for dose reduction and monitoring after initial atropinization. This may reduce anticholinergic complications while preventing the recurrence of life-threatening cholinergic symptoms.

## Conclusions

Atropine-associated delirium is an important and potentially reversible complication during the treatment of organophosphorus poisoning. Because it can closely resemble the CNS manifestations of the poisoning itself, recognition may be difficult and unnecessary atropine exposure may continue. A temporal relationship with atropine administration, improvement of the initial cholinergic manifestations, emergence of peripheral anticholinergic findings, exclusion of plausible alternative causes, and improvement following atropine reduction or withdrawal may support the diagnosis. Regular clinical reassessment and careful atropine titration are essential to minimize avoidable toxicity without compromising treatment of recurrent cholinergic manifestations.
